# Does a New Modification of the Two-Step Injection Technique for Inferior Alveolar Nerve Block Reduce Pain Compared to the Conventional Technique? A Randomized Clinical Trial

**DOI:** 10.1155/2023/5922663

**Published:** 2023-03-17

**Authors:** Amirhossein Moaddabi, Alessandra Valletta, Mohammad Koochek Dezfuli, Parisa Soltani, Saeedeh Ebrahimikiyasari, Abolfazl Hosseinnataj, Kioumars Tavakoli Tafti, Gianrico Spagnuolo

**Affiliations:** ^1^Department of Oral and Maxillofacial Surgery, Dental Research Center, School of Dentistry, Mazandaran University of Medical Sciences, Sari, Iran; ^2^Department of Neurosciences, Reproductive and Odontostomatological Sciences, University of Naples Federico II, Naples, Italy; ^3^Department of Oral and Maxillofacial Pathology, School of Dentistry, Mazandaran University of Medical Sciences, Sari, Iran; ^4^Department of Oral and Maxillofacial Radiology, Dental Implants Research Center, Dental Research Institute, School of Dentistry, Isfahan University of Medical Sciences, Isfahan, Iran; ^5^Private Practice, Sari, Iran; ^6^Department of Biostatistics, Faculty of Health, Mazandaran University of Medical Sciences, Sari, Iran; ^7^Dental Students' Research Committee, School of Dentistry, Isfahan University of Medical Sciences, Isfahan, Iran; ^8^Therapeutic Dentistry Department, Institute of Dentistry, Sechenov University, Moscow, Russia

## Abstract

**Background:**

The ability to control pain is an essential part of dental procedures and the need for optimal pain control and reduction of discomfort is the primary concern of every dentist. This study aims to compare the pain and vital signs during inferior alveolar nerve block between conventional and a new modification of the two-step injection techniques.

**Methods:**

In this institutional single-blind randomized clinical trial, attendees of dental school at Mazandaran University of Medical Sciences from February to May 2022 were included. Inclusion criteria were 20–60 years old and healthy (ASA1) individuals who were willing to participate in this study. Individuals who were taking medications affecting their understanding of pain and patients with active infections at the injection site were excluded. These individuals were divided into two groups. First, superficial anesthesia was performed and afterward, conventional and two-step injection techniques were performed. For the two-step injection method, 6 mm of the needle was injected into the mucosa and one-third of the local anesthetic solution was released from the computer-controlled injection toolkit. Afterward, a 25 mm 30-gauge needle was reinserted into the previous hole delivering the remaining local anesthetic. The pain during injection was measured by a patient-reported numerical rating scale (NRS). Moreover, vital signs were monitored immediately before and after the injection. Kolmogorov–Smirnov test, Mann–Whitney *U* test, independent *T*-test, and Fisher's exact *χ*^2^ test were performed for statistical analysis (*α* = 0.05).

**Results:**

This study involved 32 adults aged between 20 and 50 years old with 1 : 1 female/male sex distribution. The pain score was significantly higher in the conventional injection technique compared to the two-step injection technique in all sex and age groups. There were no significant differences in vital signs between the conventional and two-step injection techniques. There was no significant difference in the mean pain scores of females and males, regardless of their injection techniques.

**Conclusion:**

Utilizing the two-step injection technique in patients for inferior alveolar block reduces pain during injection without altering patients' vital signs significantly. This trial is registered with IRCT20220106053646N1.

## 1. Introduction

The ability to control pain is an essential part of dental procedures and the need for optimal pain control and reduction of discomfort is the primary concern of every dentist. Dentists throughout history have attempted to use various approaches, from alcohol to herbal remedies, to relieve patients' pain. However, the injection of local anesthetics can be a source of fear and pain and thus, patients may refuse to get injections during dental procedures or postpone their dental appointment [1]. There are various causes that lead to dental fear in patients, including previous personal and familiar experiences, personal characteristics, media illustrations of dental procedures, observation of sharp needles, and hearing the sound of dental handpieces. As a result, varied techniques have been utilized to reduce patients' pain-induced fear and anxiety, such as low-level laser therapy, superficial anesthesia, music therapy, psychosomatic methods, cold therapy of soft tissues, distracting techniques, employment of fine needles, computer-controlled local anesthetic delivery technique, and jet injection. However, all of these techniques have some restrictions and, therefore, they need to be superseded by more suitable approaches for pain reduction [2–11]. Inferior alveolar nerve block (IANB) has been widely used for the anesthesia of mandibular molar and premolar teeth during various dental procedures. In the conventional method for IANB, the clinicians use the anatomical landmarks, including the coronoid process, anterior and posterior border of the mandible, sigmoid notch, and mandibular condyle, to locate the injection site [12, 13]. The conventional technique of IANB is a challenging approach for clinicians because it requires adequate knowledge of the anatomy of jaws and for patients due to causing pain during the injection [14]. In addition to the pain caused by needle penetration and injection, the infusion of anesthetic solutions and interstitial pressure in tissue can cause a significant feeling of pressure in patients [15, 16]. Moreover, needle removal, numerous injections, inadequate needle design, and poor injection technique may lead to postoperative pain due to tissue damage [17].

The two-step injection techniques were proposed by Walton and Torabinejad [18] and Levine [19] to reduce injection pain. Compared to the conventional method, reports suggest that the two-step injection method is preferable to the conventional IANB [20]. This method involves the initial application of the anesthetic solution just under the mucosal surface. After waiting for a few minutes, the needle is inserted toward the target site and the remaining anesthetic solution is injected at the target site [20]. The choice of anesthetic is based on the surgeon's preference and the patient's medical profile. This technique is often performed by 4% prilocaine as the primary factor for superficial injection, followed by lidocaine or articaine for the anesthetic injection at the desired site in the second step [21].

Limited studies have been conducted on the evaluation of the success of the two-step injection technique for anesthesia. In the studies by Park et al. [22], Joseph et al. [23], and Rao et al. [24], the patients experienced less pain during the modified two-step injection method compared to the conventional method. In addition, the study by Sandeep et al. [25] showed that the two-step injection method significantly reduced pain in children. On the other hand, a study by Nusstein et al. [20] showed that employing this method leads to less pain only in females. Moreover, the study by Blagova and Yanev [26] demonstrated that utilizing this technique does not lead to any significant difference in pain levels compared to the conventional technique.

Due to the controversial findings of previous studies, it is essential to evaluate the two-step injection technique in different populations from different backgrounds and different age groups. This study aims to compare the pain level and vital signs of individuals in IANB using the conventional and a new modification of the two-step injection techniques.

## 2. Materials and Methods

### 2.1. Case Selection and Randomization

In this parallel single-blind randomized controlled trial, 32 individuals from all patients who attended dental school at Mazandaran University of Medical Sciences from February to May 2022 were selected. According to the sample size calculation mentioned below, the sample size of this study was calculated based on the study of Park et al. [22] as a minimum number of 12 patients for each group.

(1) $$\begin{array}{c} n=\frac{{\left(z_{1-\alpha /2}+z_{1-\beta }\right)}^{2}\times \left({\sigma_{1}}^{2}+{\sigma_{2}}^{2}\right)}{{\left(\mu_{1}-\mu_{2}\right)}^{2}}=12.7, \end{array}$$

(2) $$\begin{array}{c} \alpha =0.01,\;\beta =0.1. \end{array}$$

All procedures followed were in accordance with the principles stated in the Declaration of Helsinki “Ethical Principles for Medical Research Involving Human Subjects,” adopted by the 18th World Medical Assembly, Helsinki, Finland, June 1964, and as amended most recently by the 64th World Medical Assembly, Fortaleza, Brazil, October 2013. All procedures performed in this study were approved by the Ethical Committee of Mazandaran University of Medical Sciences (#IR.MAZUMS.REC.1400.671). Patients included in this study were informed before the examination, and an informed consent form was signed by all participants. As inclusion criteria, the individuals who were 20–60 years old, healthy (ASA1), and willing to participate in this study were recruited. For standardization of the psychological condition, which can affect individuals' pain perception [27], the General Health Questionnaire-28 (GHQ-28) was utilized. The reliability and validity of this questionnaire have been approved [28]. In addition, participants who were taking medications that alter their understanding of pain and patients with active infections at the injection site were excluded from this study [27, 29]. For the random allocation of patients into two groups, the block technique with eight blocks with a size of 4 was employed by using the random allocation software 2.0 (Mahmoud Saghaei, Isfahan, Iran) [30].

### 2.2. Preparation for Injection

**Figure 1:**
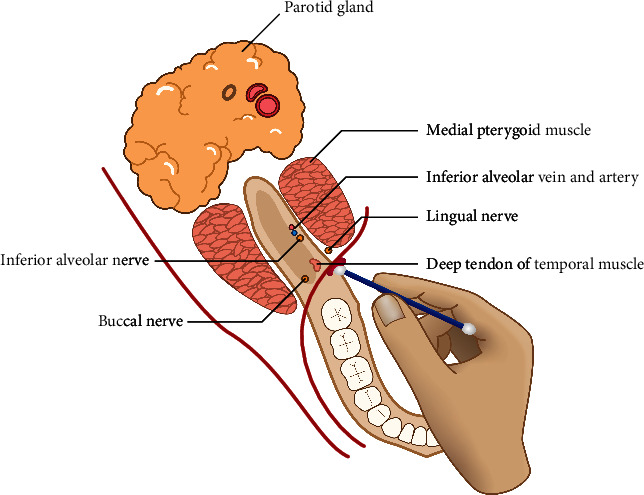
Applying topical anesthetic gel.

The patients were seated on the dental unit for 10 min to restore heart rate and blood pressure to normal levels [31]. The patients were in a semisupine position (45°) and were asked to open their mouths widely. Thereafter, oral mucosa got dried with a piece of gauze and 20% benzocaine topical anesthetic gel (Dentonics, North Carolina, USA) was applied using the applicator in the needle penetration zone for 2 min (Figure 1).

### 2.3. Injection Techniques

#### 2.3.1. Conventional Injection Technique

A 25 mm 30-gauge needle (Transcodent, Schleswig-Holstein, Germany) was used for conventional IANB injection. Moreover, a computer-controlled injection toolkit (ICT injection SE, GENOSS, Gyeonggi-do, South Korea) was utilized for injecting at different speeds (controlling the speed allows a full cartridge to be injected within 250, 120, or 50 s). The needle was inserted into the tissue by using the computer-controlled injection toolkit with the highest speed (50 s for injecting 1.8 ml of anesthetic solution). During the penetration of the needle, 0.4 ml of 2% lidocaine with 1 : 80,000 epinephrine anesthetic solution (Darou Pakhsh, Tehran, Iran) was injected. The rest of the anesthetic solution (1.4 ml) was injected after the needle reached the mandibular bone in 1 min.

#### 2.3.2. New Modification of Two-Step Injection Technique

**Figure 2:**
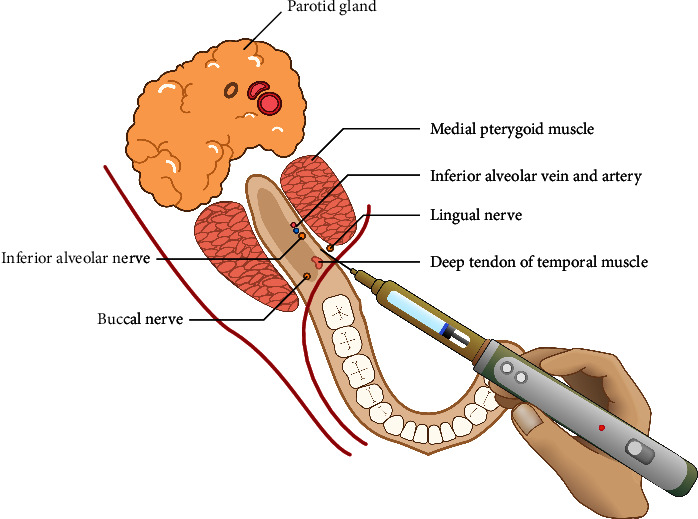
Step 1: 5 mm needle insertion into the tissue.

In this modification of the two-step injection technique, the first step of injection was operated by using a 12 mm 30-gauge dental needles (Transcodent, Schleswig-Holstein, Germany) at the lowest speed of the computer-controlled injection toolkit (250 s/1.8 ml). Because the topical anesthetic gel, which was applied before, had affected the 5 mm depth of soft tissue [16], 5 mm of the first needle was injected into the mucosa and one-third of the 2% lidocaine with 1 : 80,000 epinephrine local anesthetic solution (Darou Pakhsh, Tehran, Iran) was released (Figure 2).

**Figure 3:**
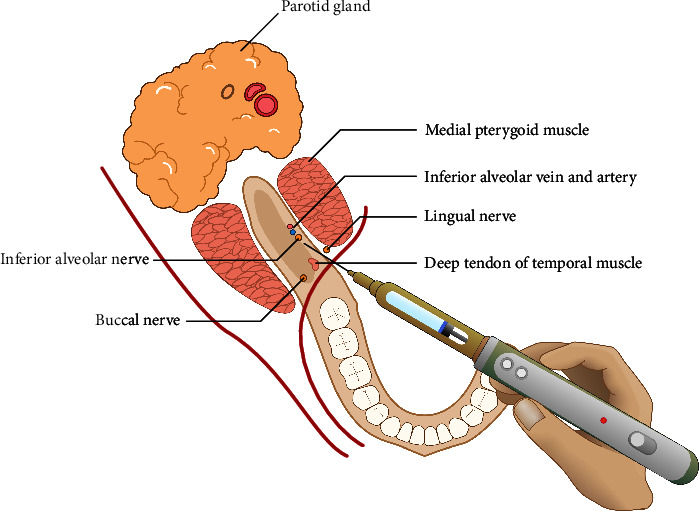
Step 2: Final injection for local anesthetic solution delivery.

After waiting for 2 min, the second needle was used, which had the same properties as the needle used for the conventional technique. The entire length of a 25 mm 30-gauge needle was reinserted into the previous short needle penetration hole at the highest speed (50 s/1.8 ml) moving to the desired area for delivering the remaining local anesthetic solution (1.2 ml) (Figure 3).

Before, during, and after the injection, patients' vital signs, including the heart rate, blood pressure, and respiratory rate, were monitored and recorded in both groups by using a vital sign monitor (CMS6000, Shenzhen, China) that was attached to the patients. These injection procedures were performed by an oral and maxillofacial surgeon with 10 years of experience. Afterward, the participants reported their pain during the injection by employing a numerical rating scale (NRS) from 0 to 10 (from 0 which indicates no pain to 10 which indicates the worst pain possible).

Because of the procedure of intervention during this study, it was not possible to make the patients and operators blind due to observing the use of a second needle. Therefore, the blinding was only applied to statistical analysis.

### 2.4. Statistical Analysis

Kolmogorov–Smirnov test, Mann–Whitney *U* test, independent *t*-test, and Fisher's exact *χ*^2^ test were performed for statistical analysis by SPSS 22 (IBM, NY, USA). *p*-Value <0.05 was considered as statistically significant.

## 3. Results

Thirty-two individuals participated in this study (16 individuals for each group). The demographic information of the individuals is shown in Table 1. The demographic variables and participants' vital signs before injection were not significantly different between the two groups (*p* > 0.05).

Utilizing an independent *t*-test showed that pain scores were significantly higher in the conventional method compared to the two-step injection technique (*p*-value <0.001) (Table 2).

There was no significant relationship between pain score and vital signs and demographic variables. However, the pain score was significantly higher in conventional techniques in all sexes, age ranges, and education levels (Table 3).

## 4. Discussion

This study investigated the self-reported pain scores and vital signs of individuals during IANB using a new modification of two-step and conventional injection techniques. The findings demonstrated that the individuals who received the conventional technique for injection experienced more pain compared to those with the two-step injection technique. In addition, the pain scores of all sex and age ranges were significantly higher in the conventional injection technique compared to the two-step injection technique. Moreover, monitoring participants' vital signs showed that there were no significant differences between individuals' vital signs before and after injection in both groups.

According to the findings of this study, the pain scores and vital signs were not significantly different between males and females, regardless of their injection techniques.

In clinical dentistry, local anesthesia is a unique combination of methodology, anesthetic substances combination, and equipment. Pain and fear during dental routine treatments and surgeries are among the most important reasons for avoiding dental procedures among patients [23]. Moreover, an anesthetic injection with little or no pain leads to less fear, which can significantly improve patient behavior during dental procedures. One of the most important aspects of dental treatments is patients being pain-free during the treatment [23]. Therefore, the application of novel techniques that can improve the patient's and physician's experience by reducing the pain of local anesthetic injection is beneficial. According to the results of this study, the individuals' pain during the two-step injection technique was significantly lower compared to the conventional technique. Thus, the two-step injection technique can be employed to reduce individuals' pain and fear during dental procedures.

The findings of this study were in agreement with the studies by Park et al. [22], Joseph et al. [23], and Rao et al. [24]. In these studies, the patients experienced significantly less pain during the modified two-step injection method compared to the conventional injection method. According to the study by Sandeep et al. [25], the two-step injection method significantly reduced pain in children compared to the traditional one-step conventional injection technique. On the other hand, Blagova and Yanev [26] postulated that there was no difference in pain score between the conventional and the modified two-step INAB injection methods. The cause of this incompatibility can be related to the difference between the devices used for anesthesia and the scale used for pain assessment in two studies. Blagova and Yanev [26] utilized a standard aspiration-type dental syringe to inject anesthesia solution and used Heft–Parker visual analog scale for pain assessment. Moreover, a study by Nusstein et al. [20] showed that employing this method leads to less pain only in females.

Because of this controversy regarding the result of reducing pain by using two-step injection techniques in the abovementioned studies, we developed a new modification of the two-step injection technique. In this new modification, we tried to reduce pain compared to the two-step injection techniques that had been used in other studies by considering effective properties such as injection speed and needle length. Penetration of the needle into the oral mucosa is the first phase of the pain experience during anesthetic injections for dental and oral treatments. Previous analysis of pain perception at the needle penetration stage showed a 14%–22% incidence of moderate to severe pain [32]; therefore, we applied a topical anesthetic gel before injection. Another factor that can significantly affect patient comfort during a local anesthetic injection is the injection speed of the solution. A study by Kanaa et al. [33] assessed the impact of injection speed on patient comfort during IANB injection and showed that a controlled injection speed reduces patients' pain and increases local anesthetic efficacy. Employing newer technologies can be lucrative in various fields of dentistry; for instance, utilizing a computer-controlled injection toolkit leads to pain reduction during injection by controlling injection speed [34–37]. As a result, we used a computer-controlled injection toolkit for this new modification. Furthermore, the study by Rao et al. [24] demonstrated that the pain of injection is related to needle characteristics such as the needle length and diameter. In addition, shorter needle length causes less pain in individuals because the needle resistance during insertion into the tissue decreases, and the needle bends less during injection [38]. Moreover, a short-length needle can potentially cause less distortion and less tissue damage during the injection procedure and decrease patients' discomfort. The higher needles gauge allows a very small amount of anesthetic solution to be injected and as a result, less soft tissue expansion occurs [24]. In the new modification of this study, unlike previous studies, 12 and 25 mm 30-gauge dental needles were utilized. Moreover, employing an ICT injection SE toolkit is a contemporary and novel methods of IANB that was used in this study. Utilizing a computer-controlled injection toolkit has received positive feedback in recent years due to its advantages such as low weight (only 72 g) which prevents early fatigue of the operators' hand and leads to easy handling, inserting and removing the anesthesia cartilage is easy, and it has three different injection speeds.

In addition, the results of this study showed that in both sexes, there was a significant difference between the pain score of conventional and the two-step injection techniques and that the pain score was reduced during the two-step injection technique. Similar to our findings, a study by Park et al. [22] demonstrated that the pain in both sexes was significantly lower with the two-step injection method than with the conventional injection method, while the study by Nusstein et al. [20] demonstrated that females experienced less pain compared to males; the results of this study showed that pain scores in males and females had no significant differences in both conventional and two-step injection groups. The conflicting results can be explained by utilizing different methods of injection in the two studies. Moreover, other studies have shown that females reported more pain than men for many types of injections, particularly during needle penetration [17, 20].

The results of this study also showed that none of the vital signs showed significant differences in the two groups of conventional and two-step injection techniques. Moaddabi et al. [39] showed that blood pressure increased after lidocaine injection; the results of this study demonstrated that this injection did not cause significant differences in participants' vital signs. However, because no study has examined the vital signs before and after injection of conventional and two-step injection techniques, it is not possible to compare this finding with other studies. Moreover, the previous studies did not use the GHQ-28 for individuals having the same pain perception and psychological condition.

The limitation of this study was the lack of the possibility of patients and operators blinding. Furthermore, another limitation of this study was the patient's lack of cooperation. Moreover, utilizing this technique can cause extra costs for patients and dentists due to using multiple needles and a computer-controlled injection toolkit.

## 5. Conclusion

Utilizing the new modification of the two-step injection technique in patients for IANB reduces pain during injection, which is not related to sex and age and does not change the patient's vital signs significantly.

## Figures and Tables

**Table 1:** Demographic information and individual's vital signs before injection.

			Injection techniques		
Variables		Total	Conventional technique	Two-step technique	*p*-Value
Sex (number)^a^	Male	16	8	8	>0.999
	Female	16	8	8	

Age range (number)^a^	20–29	7	2	5	0.329
	30–39	21	11	10	
	40–49	4	3	1	

Education (number)^a^	Bachelor's degree and below	7	3	4	0.643
	Master's degree	17	10	7	
	PhD and above	8	3	5	

Heart rate (mean)^b^		81.06	81.63	80.5	0.7
Systolic blood pressure (mean)^b^		117.19	117.5	116.88	0.705
Diastolic blood pressure (mean)^b^		77.19	78.13	76.25	0.413
Respiratory rate (mean)^b^		18.41	18.38	18.44	0.904
GHQ-28 score (mean)^b^		38.78	39.06	38.5	0.532

^a^Fisher's exact *χ*^2^ test. ^b^Independent *t*-test.

**Table 2:** Pain score in individual's variables.

				Injection techniques		
Variables		Total	*p*-Value	Conventional technique	Two-step technique	*p*-Value
Sex (mean)^a^	Male	2.19	0.579	3.88	0.50	<0.001
	Female	2.63		4.75	0.50	<0.001

Age range (mean)^a^	20–29	1.57	0.371	4.50	0.40	0.011
	30–39	2.80		4.63	0.71	<0.001
	40–49	2.40		3.83	0.25	<0.001

Education (mean)^a^	Bachelor's degree and below	1.86	0.683	4.33	0.0	0.001
	Master's degree	2.71		4.10	0.71	<0.001
	PhD and above	2.25		5.00	0.60	0.001

Heart rate (*r*)^b^		−0.12	0.503	−0.11	−0.38	–
Systolic blood pressure (*r*)^b^		−0.11	0.556	−0.34	−0.43	–
Diastolic blood pressure (*r*)^b^		−0.06	0.767	−0.31	−0.57^c^	–
Respiratory rate (*r*)^b^		0.30	0.102	0.25	−0.08	–
GHQ-28 score (*r*)^b^		0.06	0.727	−0.09	−0.06	–

^a^Independent *t*-test. ^b^Pearson correlation test. ^c^Significant *p*-value <0.05.

**Table 3:** Comparison of pain score in two groups.

Groups	Mean	Minimum–Maximum	*p*-value
Two-step injection technique	0.50	0–2	<0.001
Conventional injection technique	4.31	2–6	

## Data Availability

The datasets analyzed during this study are not publicly available but are available from the corresponding author on reasonable request.

## Abbreviations

IANB: Inferior alveolar nerve block
NRS: Numerical rating scale.
